# Supplementary material for: Differential Signaling Profiles of MC4R Mutations with Three Different Ligands
Source: Int J Mol Sci. 2020 Feb 12;21(4):1224. doi: 10.3390/ijms21041224 (PMC7072973; doi:10.3390/ijms21041224)
Supplement: Supplementary file 1 [file ijms-21-01224-s001.pdf]

Supplementary Materials/Data

Table S1. Quantification of endosomes from the confocal images. It lists the average number of endosomes measured per cell, for each mutant. Values represent quantifications of three to five independent experiments ± SEM.

MC4R mutations	<N> of endosomes/cell ± SEM
WT	17 ± 8
V103I	6 ± 2
S127L	35 ± 6
H158R	41 ± 8

Table S2. Basal (fold of WT basal), efficacy (E_{max} as fold over WT basal), potency (EC_{50} in nM) and bias values of activation of G_S signaling and β-arrestin2 recruitment of *MC4R* mutations V103I, S127L and H158R in comparison to the MC4R WT are given as ± SEM resulting from of 3 to 7 independent experiments performed in triplicates.. . Please note that the bias is the difference between two effects that result in a negative value for loss-of-function mutations. After calculation of the bias value ($10^{\Delta log(Emax/EC50)}$) the outcome of those is very close to zero and is stated as 0.00.

Signaling pathway	cAMP via G_S signaling									
MC4R mutation	basal	α-MSH			β-MSH			NDP-α-MSH		
		EC_{50}	E_{max}	bias	EC_{50}	E_{max}	bias	EC_{50}	E_{max}	bias
WT	1.00 ± 0.13	10.12 ± 3.78	23.84 ± 1.94	1.00 ± 0.00	32.70 ± 10.86	10.05 ± 0.75	1.00 ± 0.00	3.98 ± 2.17	9.52 ± 1.15	1.00 ± 0.000
V103I	1.05 ± 0.11	16.19 ± 6.21	16.91 ± 2.12	0.83 ± 0.26	101.86 ± 46.05	8.69 ± 1.10	0.28 ± 0.32	2.12 ± 0.36	6.66 ± 1.01	1.32 ± 0.510
S127L	1.01 ± 0.10	n.d.	n.d.	0.03 ± 0.06	870.23 ± 147.64***	2.90 ± 2.67*	0.01 ± 0.87	12.87 ± 8.84	3.27 ± 0.36	0.11 ± 0.415
H158R	1.43 ± 0.14 *	3.04 ± 5.18	34.38 ± 3.16*	4.80 ± 1.67 ***	29.11 ± 14.90	14.85 ± 1.27	1.66 ± 0.39	1.96 ± 0.22	12.17 ± 1.41	2.60 ± 0.534

Signaling pathway	β-arrestin2 recruitment									
MC4R mutation	basal	α-MSH			β-MSH			NDP-α-MSH		
		EC_{50}	E_{max}	bias	EC_{50}	E_{max}	bias	EC_{50}	E_{max}	bias
WT	1.00 ± 0.01	154.48 ± 0.03	2.18 ± 0.09	1.00 ± 0.00	281.20 ± 0.07	1.93 ± 0.03	1.00 ± 0.00	106.48 ± 0.00	1.91 ± 0.02	1.00 ± 0.00
V103I	0.38 ± 0.01 ***	164.51 ± 0.06***	1.71 ± 0.10*	0.74 ± 0.04 ***	243.29 ± 0.05***	1.35 ± 0.02***	0.81 ± 0.00 ***	57.99 ± 0.00***	1.34 ± 0.01***	1.29 ± 0.01 ***
S127L	0.85 ± 0.01 ***	1012.37 ± 0.51***	1.11 ± 0.02***	0.08 ± 0.04 ***	2044.70 ± 0.48***	1.01 ± 0.01***	0.07 ± 0.01 ***	54.97 ± 0.02***	1.12 ± 0.01***	1.14 ± 0.00 *
H158R	0.68 ± 0.02 ***	246.52 ± 0.07***	1.54 ± 0.10*	0.44 ± 0.04 ***	473.86 ± 0.02***	1.68 ± 0.02***	0.52 ± 0.01 ***	59.86 ± 0.002***	1.56 ± 0.01***	1.46 ± 0.01 ***

*$p \leq 0.05$, ** $p \leq 0.01$, *** $p \leq 0.001$, Statistical analysis has been performed using one-way ANOVA for basal and E_{max} value comparison to WT and two-way ANOVA for bias and EC_{50} value comparison to WT, followed by Dunnett's post-hoc test. When maximal response was lower than 25% of the maximal response of WT, values for EC_{50} and E_{max} are stated as not determinable (n.d.). Nevertheless, for the purpose of bias value calculation, EC_{50} and E_{max} were set as described in the method section.

Table S3. Basal (fold of WT basal), efficacy (E_{max} as fold over WT basal), potency (EC_{50} in nM) and bias values of activation of $G_{i/0}$ and $G_{q/11}$ signaling of *MC4R* mutations V103I, S127L and H158R in comparison to the MC4R WT are given as ± SEM resulting from of 3 to 7 independent experiments performed in triplicates. Please note that the bias is the difference between two effects that result in a negative value for loss-of-function mutations. After calculation of the bias value ($10^{\Delta log(Emax/EC50)}$) the outcome of those is very close to zero and is stated as 0.00.

Signaling pathway	PTX-sensitive cAMP via $G_{i/0}$ signaling									
MC4R mutation	basal	α-MSH			β-MSH			NDP-α-MSH		
		EC_{50}	E_{max}	bias	EC_{50}	E_{max}	bias	EC_{50}	E_{max}	bias
WT	1.18 ± 0.14	19.31 ± 6.16	39.08 ± 4.38	1.00 ± 0.00	118,10 ± 51.92	26.86 ± 2.53	1.00 ± 0.00	47.42 ± 37.15	15.82 ± 1.81	1.00 ± 0.00
V103I	1.65 ± 0.11	165.45 ± 111.21	30.17 ± 12.26	0.09 ± 0.71	41.15 ± 21.85	16.65 ± 2.19	1.78 ± 0.31	38.42 ± 25.82	8.36 ± 1.02	0.65 ± 0.19
S127L	1.57 ± 0.19	n.d.	n.d.	0.00 ± 0.22	n.d.	n.d.	0.02 ± 0.16	n.d.	n.d.	0.06 ± 1.21
H158R	1.11 ± 0.23	29.05 ± 15.40	65.65 ± 10,85	1.12 ± 0.44	50.32 ± 30.32	38.55 ± 5.90	3.37 ± 0.43 *	70.78 ± 40.90	14.34 ± 1.66	0.61 ± 0.22

Signaling pathway	PLC activation via $G_{q/11}$ signaling									
MC4R mutation	basal	α-MSH			β-MSH			NDP-α-MSH		
		EC_{50}	E_{max}	bias	EC_{50}	E_{max}	bias	EC_{50}	E_{max}	bias
WT	1.00 ± 0.17	488.87 ± 0.02	7.67 ± 1.97	1.00 ± 0.00	106.67 ± 0.04	6.58 ± 0.58	1.00 ± 0.00	30.36 ± 0.01	6.36 ± 0.58	1.00 ± 0.00
V103I	1.11 ± 0.22	89.10 ± 0.03***	6.03 ± 1.06	4.31 ± 0.19 ***	63.37 ± 0.03***	14.65 ± 1.67	3.75 ± 0.07 ***	68.19 ± 0.03***	11.03 ± 1.35	0.77 ± 0.08
S127L	0.84 ± 0.19 **	n.d.	n.d.	0.01 ± 0.09 ***	.n.d.	n.d.	0.00 ± 0.18 ***	n.d.	n.d.	0.00 ± 0.16 ***
H158R	1.37 ± 0.39	163.29 ± 0.02***	10.88 ± 2.72*	4.25 ± 0.06 ***	53.24 ± 0.02***	10.40 ± 1.95	3.17 ± 0.17 ***	39.21 ± 0.05***	9.94 ± 2.16	1.21 ± 0.20

*$p \leq 0.05$, ** $p \leq 0.01$, *** $p \leq 0.001$, Statistical analysis has been performed using one-way ANOVA for basal and E_{max} value comparison and two-way ANOVA for bias and EC_{50} value comparison, followed by Dunnett's post-hoc test; # EC_{50} was set to highest concentration when outside of tested concentration range. When maximal response was lower than 25% of the maximal response of WT, values for EC_{50} and E_{max} are stated as not determinable (n.d.). Nevertheless, for the purpose of bias value calculation, EC_{50} and E_{max} were set as described in the method section.

Table S4. Basal (fold of WT basal), efficacy (E_{max} as fold over WT basal), potency (EC_{50} in nM) and bias values of activation of $G_{12/13}$ and ERK phosphorylation of *MC4R* mutations V103I, S127L and H158R in comparison to the MC4R WT are given as ± SEM resulting from of 3 to 7 independent experiments performed in triplicates. Please note that the bias is the difference between two effects that result in a negative value for loss-of-function mutations. After calculation of the bias value ($10^{\Delta log(Emax/EC50)}$) the outcome of those is very close to zero and is stated as 0.00.

Signaling pathway	RhoA activation via $G_{12/13}$ signaling									
MC4R mutation	basal	α-MSH			β-MSH			NDP-α-MSH		
		EC_{50}	E_{max}	bias	EC_{50}	E_{max}	bias	EC_{50}	E_{max}	bias
WT	1.00 ± 0.20	77.21 ± 0.02	2.93 ± 0.47	1.00 ± 0.00	59.83 ± 0.05	2.40 ± 0.42	1.00 ± 0.00	4.68 ± 0.00	3.07 ± 0.39	1.00 ± 0.00
V103I	1.27 ± 0.21	180.59 ± 0.14***	6.09 ± 1.24*	0.59± 0.16 *	18.75 ± 0.01***	2.29 ± 0.50	3.05 ± 0.13 ***	3.01 ± 0.00	3.31 ± 0.34	1.68 ± 0.08 ***
S127L	1.12 ± 0.22	n.d.	n.d.	0.00 ± 0.25 ***	10000.00 ± 2.16*** #	0.80 ± 0.12***	0.00 ± 0.10 ***	2611.84 ± 2.13***	1.25 ± 0.20***	0.00 ± 0.09 ***
H158R	0.75 ± 0.11 **	107.47 ± 0.03***	2.82 ± 0.34	1.32 ± 0.07	39.10 ± 0.13***	2.18 ± 0.38	1.39 ± 0.03 *	1.56 ± 0.00*	1.99 ± 0.30	1.95 ± 0.08 ***

Signaling pathway	ERK phosphorylation									
MC4R mutation	basal	α-MSH			β-MSH			NDP-α-MSH		
		EC_{50}	E_{max}	bias	EC_{50}	E_{max}	bias	EC_{50}	E_{max}	bias
WT	1.00 ± 0.18	151.24 ± 0.02	7.05 ± 0.69	1.00 ± 0.00	321.15 ± 0.11	4.94 ± 0.61	1.00 ± 0.00	17.22 ± 0.01	7.47 ± 0.89	1.00 ± 0.00
V103I	1.22 ± 0.21	131.91 ± 0.02***	8.37 ± 0.62	1.21 ± 0.03	244.77 ± 0.15***	5.14 ± 0.42	1.37 ± 0.09	9.66 ± 0.00***	7.43 ± 0.55	1.77 ± 0.09 ***
S127L	1.21 ± 0.16	n.d.	n.d.	0.01 ± 0.22 ***	n.d.	n.d.	0.01 ± 0.23 ***	65.80 ± 0.01***	2.68 ± 0.27*	0.09 ± 0.06 ***
H158R	1.38 ± 0.22	273.76 ± 0.06***	10.18 ± 2.24	0.67 ± 0.07	153.71 ± 0.06***	5.55 ± 0.46	2.35 ± 0.09 ***	8.09 ± 0.00***	9.64 ± 0.51	2.75 ± 0.11 ***

*$p \leq 0.05$, ** $p \leq 0.01$, *** $p \leq 0.001$, Statistical analysis has been performed using one-way ANOVA for basal and E_{max} value comparison and two-way ANOVA for bias and EC_{50} value comparison, followed by Dunnett's post-hoc test. When maximal response was lower than 25% of the maximal response of WT, values for EC_{50} and E_{max} are stated as not determinable (n.d.). Nevertheless, for the purpose of bias value calculation, EC_{50} and E_{max} were set as described in the method section. Nevertheless, for the purpose of bias value calculation, EC_{50} and E_{max} were set as described in the method section.

Table S5. Mutation were introduced into MC4R cDNA by site-directed mutagenesis using the following primer, base pair substitution is printed in **bold.**

MC4R mutation	Mutagenesis primer	sequence
V103I	MC4R_mut_V103I _F	GGATCAGAAACCATT**A**TCATCACCCTATTAA
	MC4R_mut_V103I _R	TTAATAGGGTGATGA**T**AATGGTTTCTGATCC
S127L	MC4R_mut_S127L _F	GATAATGTCATTGACT**T**GGTGATCTGTAGCTCC
	MC4R_mut_S127L _R	GGAGCTACAGATCACC**A**AGTCAATGACATTATC
H158R	MC4R_mut_H158R _F	CTTCTATGCTCTCCAGTACC**G**TAACATTATGACAGTTAAGC
	MC4R_mut_H158R _R	GCTTAACTGTCATAATGTTA**C**GGTACTGGAGAGCATAGAAG

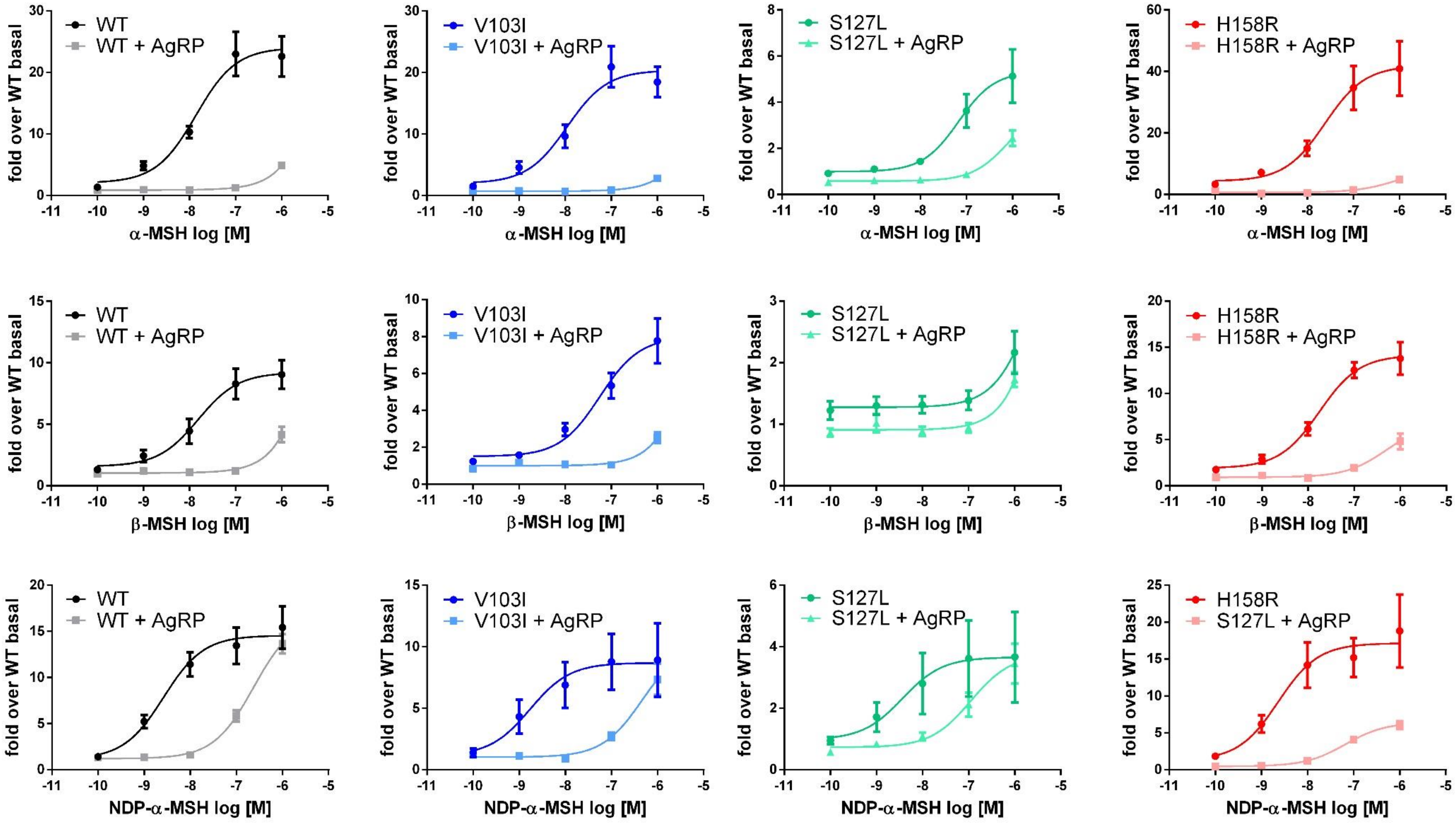

Figure S1. The antagonist AgRP decreased Gs signaling for all tested MC4R mutations and ligands. The datasets contain pooled data from three to four independent experiments each performed in triplicates. All concentration-response curves were analyzed with Graph Pad Prism 6.0 using the non-linear regression model (sigmoidal response).

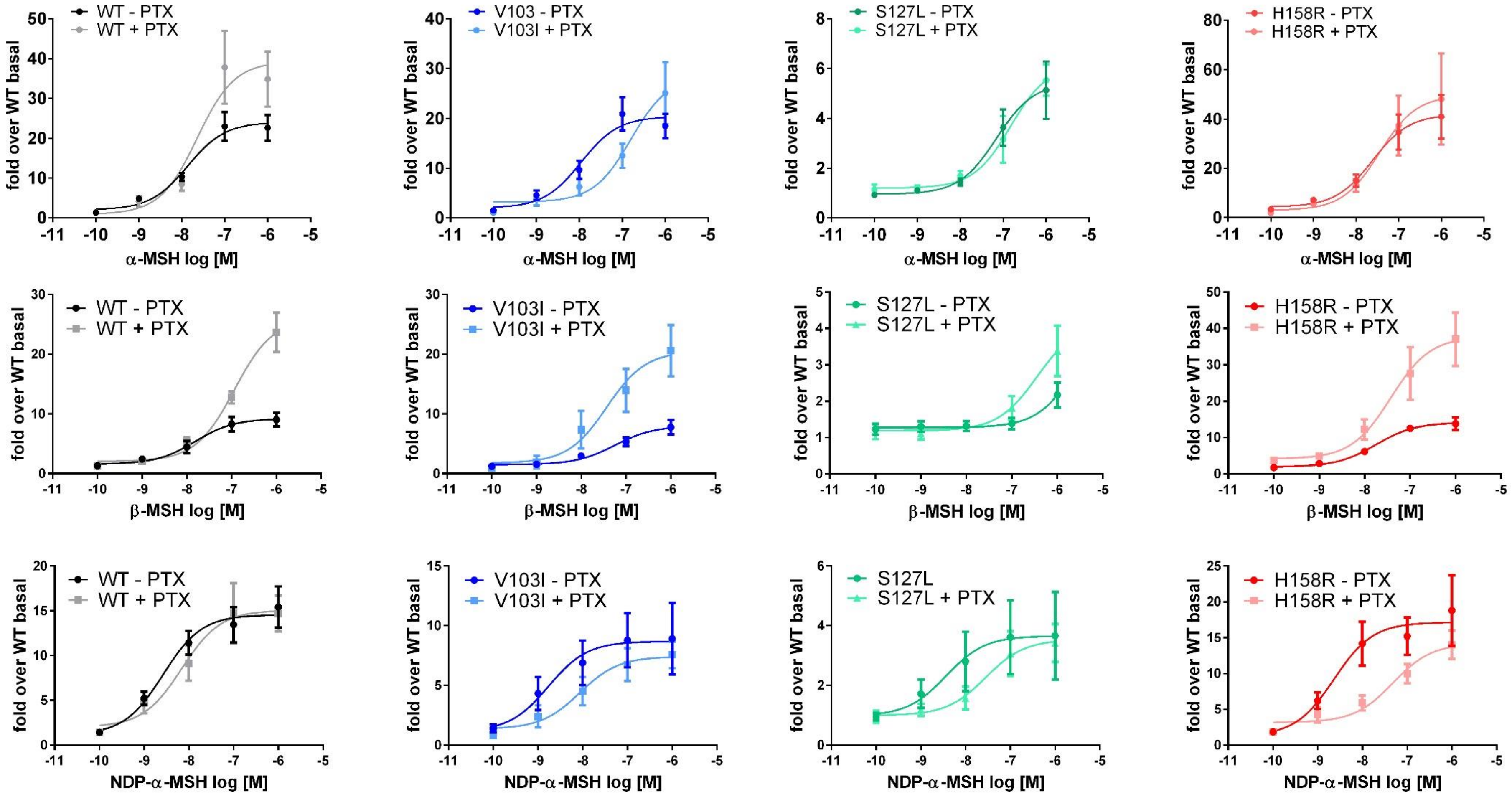

Figure S2. Contributions from $G_{i/0}$ coupling was determined by measuring the PTX-sensitive cAMP accumulation. The datasets contain pooled data from three to five independent experiments each performed in triplicates. All concentration-response curves were analyzed with Graph Pad Prism 6.0 using the non-linear regression model (sigmoidal response).
